# Liver Stiffness Variability and Limited Performance of Non-Invasive Fibrosis Scores in Hemodialysis: A Prospective Study

**DOI:** 10.3390/diagnostics16132080

**Published:** 2026-07-02

**Authors:** Karem Awad, Fadi Abu Baker, Mahmoud Foqara, Alexander Shtarkman, Abdellatif Zhalka, Tor Regev-Sadeh, Rawi Hazzan

**Affiliations:** 1 Department of Nephrology and Hypertension, Emek Medical Center, Afula 1834111, Israel; karem-awad@hotmail.co.il; 2 Department of Gastroenterology and Hepatology, Hillel Yaffe Medical Center, Hadera 38100, Israel; fadia@hymc.gov.il; 3 Rappaport Faculty of Medicine, Technion—Institute of Technology, Haifa 3525433, Israel; 4 Department of Internal Medicine B, Emek Medical Center, Afula 1834111, Israel; mahmudfuq@clalit.org.il (M.F.); zhalka_abed@hotmail.com (A.Z.); 5 Gastroenterology Department, Emek Medical Center, Afula 1834111, Israel; alex_sht@clalit.org.il; 6 Liver Clinic, Clalit Health Services, Northern Region, Nazareth 1622222, Israel; rawihazzan1@gmail.com; 7 Azrieli Faculty of Medicine, Bar-Ilan University, Safed 1311502, Israel

**Keywords:** end-stage renal disease, vibration-controlled transient elastography, hepatic congestion, ultrafiltration, fibrosis biomarkers, diagnostic accuracy

## Abstract

**Background:** Transient elastography (TE) is widely used for noninvasive assessment of liver fibrosis. In patients undergoing hemodialysis, however, liver stiffness measurements (LSM) may be affected by rapid intradialytic changes in volume status, venous congestion, and other non-fibrotic determinants. We prospectively evaluated peridialytic variability in liver stiffness and the concordance of serum fibrosis indices with elevated LSM in patients receiving maintenance hemodialysis. **Methods:** In this prospective paired pilot study, 45 adults on maintenance hemodialysis underwent LSM and controlled attenuation parameter (CAP) assessments immediately before and after a dialysis session; paired data were available for 41 patients. The Fibrosis-4 index (FIB-4) and the aspartate aminotransferase-to-platelet ratio index (APRI) were calculated from routine laboratory values. Paired comparisons, correlation analyses, and receiver operating characteristic curves were used to assess within-patient changes and the ability of serum indices to identify elevated pre-dialysis liver stiffness (LSM ≥ 8 kPa). Because no histologic or imaging reference standard for fibrosis was available, these analyses were interpreted as evidence of concordance with elevated LSM rather than as diagnostic accuracy for liver fibrosis. **Results:** Median LSM was 7.1 kPa (interquartile range [IQR] 5.2–12.1) pre-dialysis and 7.7 kPa (IQR 5.8–12.2) post-dialysis, with no significant paired change (median ΔLSM −0.2 kPa [IQR −1.1 to 1.2]; *p* = 0.898). However, the proportion with LSM ≥ 8 kPa increased from 36.6% to 46.3%, with 4 of 41 patients (9.8%) newly exceeding the threshold. CAP values showed no significant paired change (*p* = 0.511). Intradialytic weight loss was not associated with ΔLSM (rho = −0.13, *p* = 0.44). FIB-4 and APRI showed poor correlation with LSM and limited concordance with elevated LSM (area under the curve 0.553 and 0.578, respectively, with wide confidence intervals). **Conclusions:** In this exploratory hemodialysis cohort, cohort-level median LSM did not change significantly after dialysis, but clinically relevant individual-level reclassification occurred in approximately 10% of patients. Measurement timing may alter LSM-based classification, underscoring the need for dialysis-specific validation of LSM thresholds and noninvasive assessment strategies.

## 1. Introduction

Liver fibrosis represents the final common pathway of chronic liver injury arising from conditions such as chronic viral hepatitis, alcohol-related liver disease, and metabolic-associated steatotic liver disease (MASLD) [1]. Progression to cirrhosis confers substantial morbidity and mortality and remains a significant global health burden [2]. Importantly, cirrhosis is frequently diagnosed at advanced stages, and even patients with compensated disease carry a persistent annual risk of clinical decompensation [3].

Although liver biopsy has historically served as the reference standard for fibrosis assessment [4], its invasiveness and sampling limitations have accelerated the adoption of non-invasive alternatives. Among these, transient elastography (TE) has become the most widely used modality due to its accessibility, reproducibility, and favorable diagnostic performance across a range of liver diseases [5]. TE estimates liver stiffness by measuring the velocity of mechanically induced shear waves, expressed in kilopascals (kPa) [6]. Liver stiffness measurement (LSM) correlates with fibrosis stage, and values below approximately 8.0 kPa demonstrate a high negative predictive value for excluding advanced fibrosis in many clinical contexts [7]. Concurrently, the controlled attenuation parameter (CAP) enables non-invasive assessment of hepatic steatosis during the same examination [8], facilitating comprehensive evaluation of chronic liver disease in routine practice [9].

However, liver stiffness reflects not only structural fibrosis but also dynamic hemodynamic and inflammatory factors, as acknowledged in clinical practice guidance and in recent reviews of elastography-based liver assessment [10,11,12,13]. Because the liver is susceptible to changes in venous pressure and blood flow, passive hepatic congestion can transiently increase LSM independent of fibrotic remodeling. This phenomenon has been described in heart failure, tricuspid regurgitation, and constrictive pericarditis, where LSM may correlate more closely with central venous pressure than with histologic fibrosis [14,15,16,17].

Patients with end-stage renal disease (ESRD) undergoing maintenance hemodialysis represent a population in whom these confounding effects may be especially pronounced. Chronic volume overload, cardiovascular disease, and altered hemodynamics are highly prevalent, and hepatic congestion and fibrosis have been frequently documented in this group [18,19]. Although hemodialysis removes excess fluid, hepatic water content may remain unchanged or even increase due to redistribution of intravascular volume during dialysis-related circulatory stress [18]. Moreover, systemic congestion may persist before and after dialysis despite ultrafiltration [19]. Supporting the reversible contribution of hemodynamic factors, liver stiffness has been shown to decrease following kidney transplantation [20].

The performance of serum-based fibrosis indices further complicates the interpretation of liver stiffness in hemodialysis. Widely used scores such as the Fibrosis-4 index (FIB-4) and the aspartate aminotransferase–to–platelet ratio index (APRI) rely on aminotransferase levels and platelet counts, both of which are frequently altered in ESRD due to aminotransferase suppression, thrombocytopenia, and metabolic derangements [21]. As a result, the diagnostic accuracy of these indices for fibrosis assessment in hemodialysis populations remains uncertain.

Despite the increasing clinical use of transient elastography in patients with ESRD, prospective data on short-term peridialytic variability in liver stiffness are limited. Most available studies are retrospective or rely on single-time-point measurements, limiting the ability to distinguish reversible hemodynamic effects from fixed fibrosis. Whether dialysis-related fluid shifts meaningfully influence liver stiffness within a single session, and how this variability affects fibrosis classification using standard thresholds, remains incompletely defined.

Therefore, we conducted a prospective paired pilot study to evaluate LSM and CAP measurements obtained immediately before and after a single hemodialysis session. Using a within-patient design, we aimed to quantify short-term peridialytic variability in liver stiffness, examine its relationship with dialysis-related ultrafiltration, and assess the concordance of commonly used serum-based fibrosis indices with elevated pre-dialysis LSM in this high-risk population.

## 2. Methods

### 2.1. Study Design and Population

This prospective observational study was conducted over one month between 14 December 2025, and 8 January 2026, in the hemodialysis unit of a tertiary medical center. Adult patients receiving maintenance hemodialysis were consecutively screened, and 45 patients were enrolled. Patients with known right-sided heart failure were excluded a priori to minimize confounding from chronic hepatic venous congestion. The study was designed as an exploratory pilot investigation because prospective paired elastography data in maintenance hemodialysis are limited, and robust dialysis-specific effect-size estimates were unavailable for a formal power calculation. Consecutive sampling during the predefined recruitment window was therefore used to maximize feasibility and minimize selection bias.

All participants provided written informed consent before inclusion. The study protocol was approved by the Clalit EMC Institutional Ethics Committee (Approval No. 0116-25-EMC) and was conducted in accordance with the Declaration of Helsinki and the Declaration of Istanbul. All data were de-identified before analysis to ensure participant confidentiality. This study is reported in accordance with the Strengthening the Reporting of Observational Studies in Epidemiology (STROBE) guidelines.

### 2.2. Clinical Data Collection

Baseline demographic and clinical data were collected for all participants, including age, sex, dialysis vintage, and comorbid conditions. Particular attention was given to cardiometabolic comorbidities (hypertension, diabetes mellitus, dyslipidemia), cardiovascular disease, and documented chronic liver disease, including viral hepatitis, suspected or known MASLD, alcohol-related liver disease, and autoimmune or cholestatic liver disorders when recorded in the medical file.

### 2.3. Transient Elastography Measurements

Liver stiffness measurements (LSM) and controlled attenuation parameter (CAP) were assessed using vibration-controlled transient elastography (FibroScan^®^, Echosens, Paris, France). Measurements were obtained at two predefined time points during a single dialysis session: immediately before initiation of hemodialysis (pre-dialysis) and immediately after completion of the session (post-dialysis).

Experienced operators performed all examinations with more than seven years of dedicated FibroScan expertise and over 2500 prior examinations. The appropriate M or XL probe was selected in accordance with the manufacturer’s recommendations. Only examinations meeting accepted reliability criteria were included in the analysis, including acquisition of valid measurements and review of the interquartile range-to-median ratio (IQR/median) for LSM. Probe type, technical success, the number of valid measurements, and IQR/median were reviewed as quality metrics whenever available. Paired pre- and post-dialysis elastography measurements were available for 41 patients (91.1%); patients without a valid paired assessment were excluded from paired analyses.

### 2.4. Laboratory Assessments

Blood samples were obtained as part of routine clinical care and included liver enzymes [aspartate aminotransferase (AST), alanine aminotransferase (ALT)], complete blood count (including platelet count), serum albumin, and bilirubin. In accordance with routine dialysis-unit practice, available laboratory values were preferentially pre-dialysis samples; when more than one result was available, the value closest in time to the elastography assessment was used. Because blood tests were not obtained simultaneously with each FibroScan examination in all patients, this timing issue was considered when interpreting FIB-4 and APRI.

Screening for chronic liver disease included serologic testing for hepatitis B and hepatitis C. Additional laboratory testing for autoimmune liver disease was performed when clinically indicated.

### 2.5. Non-Invasive Fibrosis Scores

The Fibrosis-4 index (FIB-4) was calculated using the standard formula incorporating age, AST, ALT, and platelet count. FIB-4 values were interpreted using established thresholds: <1.3 for low probability of advanced fibrosis, 1.3–2.67 for intermediate risk, and ≥2.67 for high likelihood of advanced fibrosis.

Given the advanced age of a substantial proportion of the cohort, an age-adjusted low-risk threshold (<2.0 for patients aged ≥65 years) was additionally evaluated. The aspartate aminotransferase–to–platelet ratio index (APRI) was calculated using standard methodology and evaluated at conventional thresholds (≥0.5 and ≥1.0).

### 2.6. Study Outcomes

The primary outcome was the within-patient change in liver stiffness (ΔLSM), defined as the post-dialysis LSM minus the pre-dialysis LSM. Secondary outcomes included within-patient changes in CAP, the prevalence of elevated liver stiffness (LSM ≥ 8 kPa), threshold reclassification across 8 kPa, correlations between serum-based fibrosis scores and LSM, and the performance of FIB-4 and APRI for identifying elevated pre-dialysis LSM. The threshold of 8 kPa was selected as a pragmatic clinically used rule-out boundary for advanced fibrosis in several non-ESRD populations; however, it was not treated as a validated histologic threshold in hemodialysis.

An exploratory analysis examined the association between intradialytic ultrafiltration, estimated by net weight loss during dialysis, and within-patient change in liver stiffness.

### 2.7. Statistical Analysis

Continuous variables are presented as mean ± standard deviation (SD) or median with interquartile range (IQR), as appropriate. Categorical variables are presented as counts and percentages. Normality of continuous variables was assessed using the Shapiro–Wilk test. Paired comparisons between pre- and post-dialysis measurements (LSM and CAP) were performed using the Wilcoxon signed-rank test. Associations between liver stiffness and continuous clinical or laboratory variables, including FIB-4 and APRI, were assessed using Spearman’s rank correlation coefficient.

Receiver operating characteristic (ROC) curve analyses were performed to evaluate the ability of FIB-4 and APRI to identify elevated liver stiffness, using pre-dialysis LSM ≥ 8 kPa as the reference outcome. Because liver biopsy, magnetic resonance elastography, and other independent fibrosis reference standards were unavailable, the ROC analyses were interpreted strictly as concordance with elevated LSM rather than as diagnostic accuracy for histologic liver fibrosis. Area under the curve (AUC) values with 95% confidence intervals (CIs) were calculated, and the width of the CIs was considered when interpreting clinical utility.

The association between intradialytic weight loss and within-patient change in liver stiffness was assessed using Spearman correlation. All statistical tests were two-sided, and *p*-values < 0.05 were considered statistically significant. Statistical analyses were performed using R statistical software (version 4.x; R Foundation for Statistical Computing, Vienna, Austria).

## 3. Results

### 3.1. Study Population

A total of 45 patients receiving maintenance hemodialysis were included in the study, of whom 41 (91.1%) had paired pre- and post-dialysis transient elastography measurements available for analysis (Table 1). The cohort was elderly, with a mean age of 70.0 ± 15.8 years, and predominantly male (36/45, 80.0%). Most patients were of Jewish ethnicity (28/45), followed by Arab patients (11/45), while ethnicity was not clearly defined in a minority of cases (6/45).

The burden of cardiometabolic comorbidity was substantial. Hypertension was present in 36 patients (80.0%), diabetes mellitus in 21 (46.7%), and dyslipidemia in 24 (53.3%). Ischemic heart disease was documented in 25 patients (55.6%), reflecting a high prevalence of established cardiovascular disease in this hemodialysis population.

With respect to viral hepatitis status, no patients were positive for hepatitis B surface antigen (HBsAg; 0/45, 0.0%), while hepatitis C antibody positivity was observed in 2 patients (4.4%). Baseline liver biochemistry and related parameters were generally low, consistent with the biochemical profile commonly observed in end-stage kidney disease. Median aspartate aminotransferase (AST) was 13.0 IU/L (IQR 11.0–18.0) and median alanine aminotransferase (ALT) was 10.0 IU/L (IQR 8.0–16.0). Median serum albumin was 3.62 g/dL (IQR 3.40–3.91), and median total bilirubin was 0.51 mg/dL (IQR 0.44–0.66). Non-invasive fibrosis indices were modest overall, with a median FIB-4 score of 1.45 (IQR 0.96–1.96) and a median APRI of 0.20 (IQR 0.10–0.20). Thus, overt viral hepatitis was uncommon in this cohort. No participant had active hepatitis B infection, and only two had evidence of prior or current HCV exposure. Other chronic liver disease diagnoses were not sufficiently frequent to support disease-specific subgroup analyses; therefore, the cohort should be interpreted primarily as a maintenance hemodialysis population undergoing LSM assessment rather than as one enriched for established chronic liver disease.

### 3.2. Liver Stiffness Before and After Hemodialysis

Median LSM was 7.1 kPa (IQR 5.2–12.1) before dialysis and 7.7 kPa (IQR 5.8–12.2) after dialysis (Table 2). There was no evidence of a systematic within-patient change in LSM following dialysis (median paired difference post–pre −0.2 kPa [IQR −1.1 to 1.2]; Wilcoxon signed-rank test *p* = 0.898). The apparent increase in unpaired cohort medians and the negative median paired difference are not contradictory, because medians of marginal distributions and the median of within-patient paired differences need not be identical.

**Figure 1:**
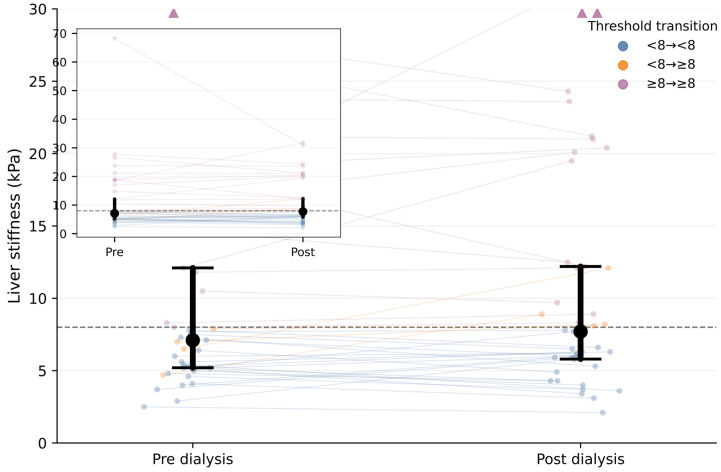
Within-patient changes in liver stiffness before and after hemodialysis—paired liver stiffness measurements before and after hemodialysis in 41 patients. Individual trajectories are shown, with medians and interquartile ranges overlaid. The dashed line indicates the 8 kPa threshold. Colors reflect the threshold transition status across dialysis. An inset displays the full LSM range.

Despite stable central tendency at the cohort level, the proportion of patients with LSM ≥ 8 kPa increased from 36.6% (15/41) pre-dialysis to 46.3% (19/41) post-dialysis. Reclassification across this threshold was attributable to 4 of 41 patients (9.8%) shifting from <8 to ≥8 kPa, with no patients moving in the opposite direction. Individual paired trajectories demonstrated bidirectional heterogeneity (Figure 1). Clinically, such reclassification could influence decisions regarding hepatology referral, repeat fibrosis assessment, surveillance planning, or additional etiologic workup if LSM is interpreted without accounting for the timing of dialysis.

### 3.3. CAP Before and After Hemodialysis

Among the 41 patients with paired CAP measurements, median CAP was 222 dB/m (IQR 167–308) before dialysis and 231 dB/m (IQR 184–272) after dialysis. There was no evidence of a systematic within-patient change in CAP across the dialysis session. The median paired difference (post–pre) was −4 dB/m (IQR −42 to 50), and the Wilcoxon signed-rank test indicated no statistically significant shift following dialysis (*p* = 0.511).

### 3.4. Performance of Non-Invasive Fibrosis Scores

Among 42 hemodialysis patients with pre-dialysis transient elastography available, 15 (35.7%) had an LSM ≥ 8 kPa; among the 41 patients with paired post-dialysis measurements, the corresponding prevalence increased to 46.3%. Median FIB-4 was 1.45 (IQR 0.96–1.96), and median APRI was 0.20 (IQR 0.10–0.20). Neither FIB-4 nor APRI demonstrated a meaningful monotonic association with liver stiffness at either time point (Spearman ρ range −0.078 to 0.203; all *p* > 0.20).

**Figure 2:**
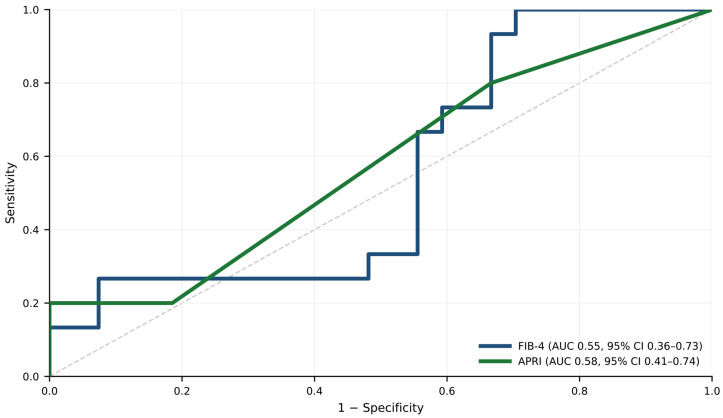
Receiver operating characteristic analysis of FIB-4 and APRI for the identification of elevated liver stiffness in hemodialysis patients. Receiver operating characteristic curves for FIB-4 and APRI in identifying elevated liver stiffness (LSM ≥ 8 kPa) using pre-dialysis transient elastography as the reference standard (*n* = 42). AUCs with 95% confidence intervals are shown. The dashed line indicates chance performance.

In receiver operating characteristic analyses using pre-dialysis LSM ≥ 8 kPa as the reference outcome (*n* = 42), both serum-based fibrosis scores showed poor concordance with elevated LSM rather than validated diagnostic accuracy for histologic fibrosis (Figure 2). FIB-4 yielded an AUC of 0.553 (95% CI 0.363–0.731), and APRI yielded an AUC of 0.578 (95% CI 0.407–0.742). These wide confidence intervals indicate substantial statistical imprecision and mean that clinically meaningful performance cannot be reliably excluded or confirmed in this pilot cohort.

Cutoff-based analyses were consistent with the ROC findings and are summarized in Table 3. The conventional FIB-4 rule-out threshold (<1.3) demonstrated moderate sensitivity (66.7%) but low specificity (44.4%), resulting in modest negative predictive value pre-dialysis (70.6%) and weaker performance post-dialysis (52.9%). In contrast, the standard rule-in threshold (FIB-4 ≥ 2.67) was particular (92.6% pre-dialysis; 95.5% post-dialysis) but poorly sensitive (26.7% and 26.3%, respectively). Application of an age-adjusted low-risk threshold (FIB-4 <2.0 in patients aged ≥65 years) reclassified 11 individuals as low risk without upward reclassification.

APRI thresholds similarly showed limited clinical utility. APRI ≥0.5 and ≥1.0 demonstrated very high specificity (100%) but extremely low sensitivity (20.0% and 6.7%, respectively), with unstable positive predictive values due to small numbers of positive cases. Overall, both serum-based indices failed to reliably distinguish patients with elevated liver stiffness in this hemodialysis cohort.

### 3.5. Ultrafiltration and Liver Stiffness Dynamics

**Figure 3:**
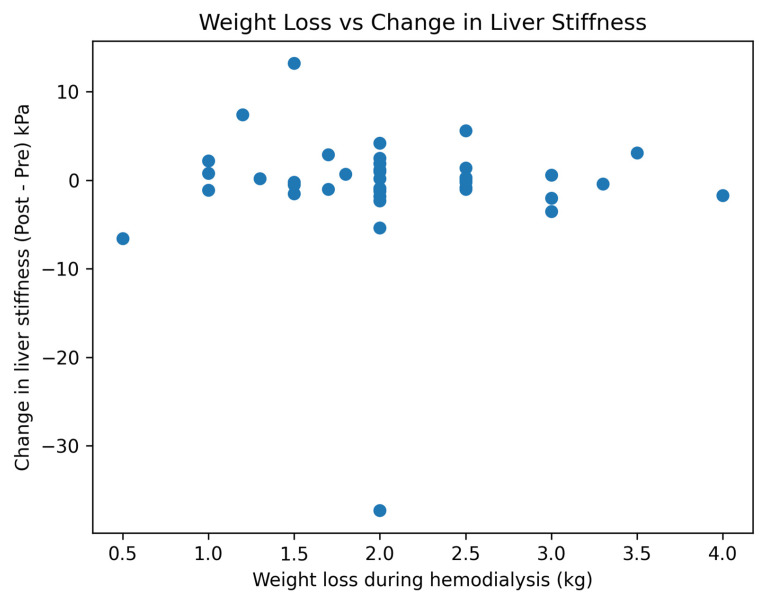
Relationship between intradialytic weight loss and change in liver stiffness. Scatter plot showing the association between ultrafiltration-related weight loss during hemodialysis (kg) and the change in liver stiffness (ΔLSM, post–pre; LSM = liver stiffness measurement). Each point represents an individual patient. No significant correlation was observed (Spearman correlation).

Mean intradialytic weight loss was 2.10 +/− 0.70 kg. There was no evidence of an association between ultrafiltration-related weight loss and within-patient change in liver stiffness (ΔLSM = post–pre; Spearman rho = −0.13, *p* = 0.44; *n* = 40) (Figure 3). Changes in liver stiffness were heterogeneous and were not explained by the magnitude of fluid removal. Because direct congestion or hemodynamic markers were not collected, the roles of venous congestion, hepatic blood-volume redistribution, and intradialytic circulatory stress should be considered mechanistic hypotheses rather than demonstrated mechanisms.

## 4. Discussion

In this prospective paired pilot study of patients receiving maintenance hemodialysis, we observed clinically relevant individual-level variability in LSM within a single dialysis session, despite no statistically significant shift in cohort-level central tendency. Median LSM was 7.1 kPa pre-dialysis and 7.7 kPa post-dialysis (paired *p* = 0.898); however, 9.8% of patients crossed the pragmatic ≥8 kPa threshold following dialysis, increasing the apparent prevalence of elevated LSM from 36.6% to 46.3%. These data should not be interpreted as evidence of an acute change in fibrosis. Rather, they highlight a dissociation between population-level stability and individual-level LSM reclassification, suggesting that measurement timing may affect clinical interpretation in hemodialysis patients.

Our findings extend earlier observations that hemodialysis can influence LSM, including the study by Kellner et al. [21] and more recent dynamic LSM data in hemodialysis patients [22]. Consistent with evidence from congestion-related states and acute inflammatory liver injury, elastography should be viewed as a composite signal influenced by fibrosis, inflammation, hepatic blood volume, sinusoidal pressure, and venous congestion, rather than fibrosis alone [11,12,13,14,15,16,17,23,24]. The bidirectional changes observed in our cohort support the need for cautious interpretation and standardized timing, but the absence of direct hemodynamic measurements prevents definitive attribution to a specific mechanism.

Recent real-world and comparative elastography data further support the need for context-specific interpretation of LSM. In a large VCTE cohort of more than 7000 patients, Lazar et al. demonstrated the broad clinical utility of VCTE in fibrosis staging across diverse liver disease settings [25]. In parallel, Losurdo et al. reported substantial but imperfect agreement between transient elastography and point shear-wave elastography, emphasizing that liver stiffness is a technique-dependent quantitative biomarker rather than a direct histologic diagnosis [11]. These observations are particularly relevant in hemodialysis, where acute volume and hemodynamic shifts may further affect LSM interpretation.

Notably, changes in LSM were not explained by ultrafiltration-related fluid removal. Despite a mean intradialytic weight loss of 2.10 +/− 0.70 kg, weight loss was not associated with within-patient ΔLSM (Spearman rho = −0.13, *p* = 0.44). This finding suggests that simple net fluid removal may be insufficient to predict LSM changes. One possible explanation is that LSM may reflect hepatic venous pressure, systemic congestion, and vascular redistribution more than absolute weight change; however, because echocardiographic parameters, natriuretic peptides, inferior vena cava measurements, and right-sided filling-pressure surrogates were not collected, this interpretation remains hypothesis-generating.

A second clinically relevant finding is the limited utility of commonly used serum-based fibrosis indices for identifying elevated LSM in this population. Neither FIB-4 nor APRI demonstrated meaningful monotonic associations with LSM at either time point, and ROC analyses using pre-dialysis LSM ≥ 8 kPa as the reference outcome showed poor concordance with wide confidence intervals. Because LSM was itself the outcome under evaluation and no independent fibrosis reference standard was available, these ROC analyses cannot establish diagnostic accuracy for advanced fibrosis. They instead demonstrate that aminotransferase- and platelet-based scores do not reliably track elevated LSM in this peridialytic setting. Recent evidence in hemodialysis patients with chronic viral hepatitis showed that FIB-4 was independently associated with 5-year all-cause mortality, supporting its potential prognostic role in selected dialysis populations. However, this does not establish diagnostic accuracy for liver fibrosis. Our findings extend this literature by showing poor concordance between FIB-4 and APRI and elevated pre-dialysis LSM, emphasizing that prognostic utility and fibrosis discrimination should not be considered interchangeable in ESRD [26].

The stability of controlled attenuation parameter measurements further informs interpretation. CAP values remained stable across dialysis sessions, in contrast to the variability observed in liver stiffness. This divergence suggests that elastography-derived metrics may differ in their sensitivity to hemodynamic perturbations versus structural parenchymal features, consistent with observations from large hemodialysis cohorts assessed using transient elastography [12]. Together, these findings imply that peridialytic physiology may disproportionately influence stiffness-based fibrosis classification, whereas CAP may be less susceptible to acute intradialytic shifts, although this warrants validation.

This study has several strengths. The prospective paired design with immediate pre- and post-dialysis assessments enables within-subject evaluation of peridialytic variability while minimizing confounding from inter-individual differences. Measurements were performed under standardized conditions by experienced operators using accepted reliability criteria, and the integration of elastography with serum-based indices provides clinically actionable evidence regarding real-world noninvasive assessment strategies in a population in whom liver biopsy is frequently impractical.

Several limitations should be considered when interpreting these findings. First, the study was conducted at a single center with a modest sample size and should be considered exploratory; this limited precision for subgroup analyses and resulted in wide confidence intervals for AUC estimates. Second, a liver biopsy, magnetic resonance elastography, or another independent fibrosis reference standard was not available. Therefore, elevated LSM cannot be definitively attributed to fibrosis, and LSM ≥ 8 kPa should be interpreted as a pragmatic LSM threshold rather than a histologic diagnosis. Third, detailed hemodynamic characterization (e.g., echocardiography, BNP/NT-proBNP, inferior vena cava measurements, central venous pressure surrogates, or right-sided filling pressures) were not systematically collected, limiting mechanistic inference. Fourth, laboratory values were selected based on their proximity to elastography and were generally routine pre-dialysis tests rather than simultaneous samples, which may introduce measurement misalignment in FIB-4 and APRI. Fifth, although experienced operators applied examination reliability criteria, complete reporting of probe type and quality metrics was limited by the available data structure. Finally, our analysis focused on acute peridialytic variability and does not address longitudinal trajectories, reproducibility across multiple dialysis sessions, or the prognostic implications of LSM reclassification.

In conclusion, maintenance hemodialysis was not associated with a significant cohort-level median change in LSM, but approximately 10% of patients crossed a pragmatic elevated-stiffness threshold after dialysis. These findings suggest that LSM-based classification in hemodialysis may be sensitive to measurement timing and individual-level physiological variation. Multicenter prospective studies incorporating independent fibrosis reference standards, standardized pre- versus post-dialysis timing, repeated-session reproducibility, and complementary hemodynamic profiling are needed to define dialysis-specific thresholds and clinical pathways.

## 5. Conclusions

Maintenance hemodialysis may be associated with clinically relevant short-term individual-level variability in LSM, even when median cohort-level values remain stable. Conventional FIB-4 and APRI showed limited concordance with elevated LSM in this exploratory cohort. Dialysis-related timing may influence LSM-based classification when standard thresholds are applied, supporting the need for dialysis-specific validation of non-invasive liver assessment strategies.

## Figures and Tables

**Table 1:** Baseline characteristics of patients undergoing maintenance hemodialysis (*N* = 45).

Variable	Value
Age, years, mean ± SD	70.0 ± 15.8
Male sex, *n* (%)	36 (80.0)
Diabetes mellitus, *n* (%)	21 (46.7)
Hypertension, *n* (%)	36 (80.0)
Hyperlipidemia, *n* (%)	24 (53.3)
Cardiovascular disease, *n* (%)	25 (55.6)
Chronic lung disease, *n* (%)	9 (20.0)
AST, IU/L, median (IQR)	13 (11–18)
ALT, IU/L, median (IQR)	10 (8–16)
Platelet count, ×10^3^/µL, median (IQR)	209 (166–248)
FIB-4, median (IQR)	1.45 (0.96–1.96)
APRI, median (IQR)	0.20 (0.10–0.20)
HBsAg positive, *n* (%)	0 (0.0)
HCV antibody positive, *n* (%)	2 (4.4)
HBsAb positive, *n* (%)	37 (82.2)
Weight loss during hemodialysis, kg, mean ± SD	2.10 ± 0.69

ALT = alanine aminotransferase; APRI = AST to Platelet Ratio Index; AST = aspartate aminotransferase; FIB-4 = Fibrosis-4 index; HBsAb = hepatitis B surface antibody; HBsAg = hepatitis B surface antigen; HCV = hepatitis C virus; IQR = interquartile range; SD = standard deviation.

**Table 2:** Paired liver stiffness and controlled attenuation parameter measurements before and after hemodialysis (*n* = 41).

Variable	Before Hemodialysis	After Hemodialysis	Δ Change (After–Before)	*p*-Value
Liver stiffness, kPa, median (IQR)	7.1 (5.2–12.1)	7.7 (5.8–12.2)	−0.2 (−1.1 to 1.2)	0.898
CAP, dB/m, median (IQR)	222 (167–308)	231 (184–272)	−4 (−42 to 50)	0.511

CAP = controlled attenuation parameter; kPa = kilopascal; dB/m = decibels per meter; IQR = interquartile range.

**Table 3:** Exploratory performance of serum-based fibrosis-score cutoffs for identifying elevated pre-dialysis liver stiffness (LSM ≥ 8 kPa).

Index and Cutoff	Reference Outcome	Performance	Interpretation
FIB-4 ≥ 1.3	Pre-HD LSM ≥ 8 kPa	Sensitivity 66.7%; specificity 44.4%; PPV 40.0%; NPV 70.6%; accuracy 52.4%	Low specificity; limited rule-out value
FIB-4 ≥ 2.67	Pre-HD LSM ≥ 8 kPa	Sensitivity 26.7%; specificity 90.0%; PPV 57.1%; NPV 71.1%; accuracy 68.9%	Specific but poorly sensitive
FIB-4 ≥ 2.67	Post-HD LSM ≥ 8 kPa	Sensitivity 26.3%; specificity 92.3%; PPV 71.4%; NPV 63.2%; accuracy 64.4%	Similar post-HD pattern
Age-adjusted FIB-4 (<2.0 low risk if age ≥ 65)	Descriptive reclassification	Reclassified 11 additional patients as low risk; no upward reclassification	Age adjustment changes risk categorization.
APRI ≥ 0.5	Pre-HD LSM ≥ 8 kPa	Sensitivity 20.0%; specificity 100%; PPV 100%; NPV 69.2%; accuracy 71.4%	Very low sensitivity
APRI ≥ 1.0	Pre-HD LSM ≥ 8 kPa	Sensitivity 6.7%; specificity 100%; PPV 100%; NPV 65.9%; accuracy 66.7%	Very low sensitivity

PPV = positive predictive value; NPV = negative predictive value; HD = hemodialysis; LSM = liver stiffness measurement. Values should be interpreted as exploratory concordance with LSM ≥ 8 kPa rather than diagnostic accuracy for histological fibrosis.

## Data Availability

The raw data supporting the conclusions of this article will be made available by the authors on request.

## Abbreviations

ALT	Alanine aminotransferase
APRI	Aspartate aminotransferase–to–platelet ratio index
AST	Aspartate aminotransferase
CAP	Controlled attenuation parameter
ESRD	End-stage renal disease
FIB-4	Fibrosis-4 index
IQR	Interquartile range
LSM	Liver stiffness measurement
ROC	Receiver operating characteristic
TE	Transient Elastography
HBsAg	Hepatitis B surface antigen
HBsAb	Hepatitis B surface antibody
HCV	Hepatitis C virus
HD	Hemodialysis
kPa	Kilopascal
PPV	Positive predictive value
NPV	Negative predictive value
SD	Standard deviation
